# Energy Footprint and Reliability of IoT Communication Protocols for Remote Sensor Networks

**DOI:** 10.3390/s25196042

**Published:** 2025-10-01

**Authors:** Jerzy Krawiec, Martyna Wybraniak-Kujawa, Ilona Jacyna-Gołda, Piotr Kotylak, Aleksandra Panek, Robert Wojtachnik, Teresa Siedlecka-Wójcikowska

**Affiliations:** 1 Faculty of Mechanical and Technological, Warsaw University of Technology, 00-661 Warsaw, Poland; jerzy.krawiec@pw.edu.pl (J.K.); martyna.wybraniak@pw.edu.pl (M.W.-K.); ilona.golda@pw.edu.pl (I.J.-G.); robert.wojtachnik@pw.edu.pl (R.W.); teresa.siedlecka@pw.edu.pl (T.S.-W.); 2 Faculty of Transport, Warsaw University of Technology, 00-661 Warsaw, Poland

**Keywords:** sensor networks, energy consumption, communication protocols, IoT, UAV, RS-IoT, reliability, maintainability

## Abstract

Excessive energy consumption of communication protocols in IoT/IIoT systems constitutes one of the key constraints for the operational longevity of remote sensor nodes, where radio transmission often incurs higher energy costs than data acquisition or local computation. Previous studies have remained fragmented, typically focusing on selected technologies or specific layers of the communication stack, which has hindered the development of comparable quantitative metrics across protocols. The aim of this study is to design and validate a unified evaluation framework enabling consistent assessment of both wired and wireless protocols in terms of energy efficiency, reliability, and maintenance costs. The proposed approach employs three complementary research methods: laboratory measurements on physical hardware, profiling of SBC devices, and simulations conducted in the COOJA/Powertrace environment. A Unified Comparative Method was developed, incorporating bilinear interpolation and weighted normalization, with its robustness confirmed by a Spearman rank correlation coefficient exceeding 0.9. The analysis demonstrates that MQTT-SN and CoAP (non-confirmable mode) exhibit the highest energy efficiency, whereas HTTP/3 and AMQP incur the greatest energy overhead. Results are consolidated in the ICoPEP matrix, which links protocol characteristics to four representative RS-IoT scenarios: unmanned aerial vehicles (UAVs), ocean buoys, meteorological stations, and urban sensor networks. The framework provides well-grounded engineering guidelines that may extend node lifetime by up to 35% through the adoption of lightweight protocol stacks and optimized sampling intervals. The principal contribution of this work is the development of a reproducible, technology-agnostic tool for comparative assessment of IoT/IIoT communication protocols. The proposed framework addresses a significant research gap in the literature and establishes a foundation for further research into the design of highly energy-efficient and reliable IoT/IIoT infrastructures, supporting scalable and long-term deployments in diverse application environments.

## 1. Introduction

IoT/IIoT now powers domains from smart homes to agriculture and increasingly underpins large-scale remote-sensing (RS-IoT) systems that monitor forests, oceans, and the atmosphere, yet radio traffic is far costlier than sensing or computing, dominantly drains batteries, making energy efficiency a co-equal goal with throughput, latency, and reliability [1]. This pressure grows as billions of battery-powered nodes proliferate, including harsh-environment RS motes, and “smart” radios draw high idle currents; optimizing the communication stack therefore offers the fastest energy return [2]. Protocol choice is central: MQTT (pub/sub), CoAP (REST-like), AMQP (queued, reliable), and omnipresent HTTP differ in header size, state complexity, and security load, and the trade-off shifts with star, mesh, cellular, or long-range remote-sensing topologies. Yet literature remains siloed: many surveys focus on a single vertical or on Layer-2 aspects, seldom quantifying end-to-end power. Limited head-to-head tests show CoAP is most frugal, MQTT beats HTTP, while AMQP buys stronger guarantees at higher cost; still, fewer than a dozen studies compare more than two protocols with statistical depth [3].

This article closes that gap by comparing the energy consumption of the most representative wired (MQTT, MQTT-SN, CoAP, AMQP, HTTP 1/2/3) and wireless (LoRaWAN, Sigfox, NB-IoT, LTE-M, BLE, ZigBee, Wi-Fi, 6LoWPAN, Wi-SUN) stacks used in IoT/IIoT and, by extension, RS-IoT deployments. The selection of “representative” protocols was guided by their relevance to real-world deployments across both industrial, consumer, and remote-sensing contexts. The final set reflects a balance between widespread legacy standards, emerging low-power technologies, and communication models suited for resource-constrained edge nodes. The rationale for adopting these criteria was to ensure that the evaluation captures both widely deployed standards and emerging low-power technologies, while covering different communication paradigms and infrastructures relevant to RS-IoT applications [4]. The choice criteria include:

- broad adoption in the field or industry-standard status,
- diversity in architectural paradigms (publish-subscribe, REST, queue-based, mesh, client-server),
- coverage across both wired and wireless infrastructures,
- support for constrained hardware and energy-aware operation,
- maturity of the ecosystem and protocol stack implementations.

Table 1 summarizes the selected representative communication protocols considered in this study.

We adopt a dual methodology: laboratory measurements quantify joule-per-payload costs for wired protocols, while a rigorously scored qualitative matrix ranks wireless technologies whose reproducible lab tests are hindered by spectrum licensing or infrastructure constraints. A Unified Comparative Method normalizes these inputs onto a common 0–1 scale, interpolates sparse datapoints, and aggregates five independent campaigns under a sensitivity-analysed weighting scheme. Key outputs include:

- the largest open dataset of energy traces for application-layer protocols under harmonized traffic patterns,
- a transparent weighting framework that yields a Spearman ρ > 0.9 across integration paths,
- actionable heuristics linking protocol choice to duty cycle, radio class, and QoS tier.

By aligning quantitative evidence with qualitative insights, the study equips practitioners with a replicable, technology-agnostic benchmark for selecting energy-optimal communication protocols and, by extension, for building sustainable IoT/IIoT infrastructures [13,14].

In light of these issues, the article is structured into six sections. Section 1 outlines key challenges related to protocol choice, focusing on energy efficiency and system reliability. Section 2 reviews related literature and identifies research gaps as well as evaluates protocols in terms of maintenance and reliability. Section 3 presents the methods adopted and a standardized measure. Section 4 discusses the experiments and results of a quantitative comparison of wired and wireless protocols, while Section 5 presents the results of the standard method, as well as an industry classification of protocol energy profiles and design guidelines for practitioners. The article concludes with key findings and directions for future work.

## 2. Related Works

### 2.1. Selection of Communication Protocols in the Literature Due to Energy Consumption

A consistent pattern emerges from early bilateral evaluations: publish/subscribe architectures impose a significantly lower energy load on IoT devices than request/response models. In a gateway-level experiment, MQTT outperformed HTTP by reducing joule-per-payload consumption by 35–60%, assuming equivalent traffic conditions [15]. This result highlights the potential of broker-based systems to optimize energy use when message overhead is minimized.

Building on this result, an oscilloscope-based study expanded the comparison matrix to include MQTT, CoAP, AMQP, HTTP/1, and HTTP/2 [16]. The results reinforced the earlier ranking, identifying CoAP as the most economical protocol, closely followed by MQTT. In contrast, AMQP exhibited the highest energy cost, attributed to its verbose framing and the need for mandatory acknowledgements.

Taken together, studies [15,16] demonstrate that protocols with compact headers and stateless client design are systematically more energy-efficient in typical gateway communication scenarios. These trends remain consistent across various hardware platforms, from ARM Cortex-M microcontrollers to SBC-class devices, suggesting architectural independence of the observed energy hierarchy.

### 2.2. Impact of Protocol Evolution (HTTP1-3, MQTT-MQTT-SN)

Protocol lineage alone does not ensure improved energy performance. When examining three generations of HTTP under identical machine-to-machine workloads, HTTP/3 exhibited higher energy consumption than HTTP/2 and HTTP/1, despite employing the more efficient QUIC handshake. The underlying cause was a greater encryption overhead per datagram, which offset any potential benefits from transport-layer optimization [17].

In parallel, attention has turned to pub/sub evolution. A simulation campaign using COOJA and Powertrace compared CoAP and MQTT-SN on resource-constrained motes [18]. The results showed nearly identical total energy consumption, yet MQTT-SN reduced radio energy use by approximately 12%, thanks to its topic-ID mechanism that eliminates the need for persistent client state.

These findings suggest that within-family protocol enhancements (e.g., MQTT → MQTT-SN) can deliver tangible energy savings, especially by reducing radio transmission time and client complexity, while cross-family upgrades (e.g., HTTP/1 → HTTP/3) may introduce unintended inefficiencies due to encryption demands.

To extend the analysis beyond point-to-point settings, a dedicated multihop study revisited CoAP and MQTT-SN under identical mesh topologies [19]. While the total transmission energy differed by less than 5%, a key distinction emerged in memory usage: MQTT-SN required 30% less SRAM than CoAP, a decisive advantage for deeply embedded nodes with strict resource budgets. Such savings are especially relevant in dense sensor networks, where both battery life and memory constraints directly impact network scalability.

This pattern is consistent with the earlier simulation findings in [18], where MQTT-SN’s stateless architecture translated into reduced radio energy usage. Together, these studies show that MQTT-SN offers not only competitive energy performance but also a significantly smaller memory footprint, which may be crucial for battery-operated mesh devices.

### 2.3. Synthesis and Research Gap

Collectively, works [15,16,17,18,19,20,21,22] quantify piecewise energy trade-offs, whereas studies [23,24,25,26] expose system-level levers such as RIS tuning or RRC state depth. Yet two voids remain:

- Cross-layer integration. No dataset correlates application-layer joule profiles with underlying radio duty-cycle governance across both wired and wireless tiers in the same experiment.
- Unified metric. Rankings differ because authors average over disparate payload sizes, QoS levels, or link budgets; a normalized, sensitivity-checked score is missing.

The present work addresses these gaps by:

- measuring six wired protocols on physical SBC gateways,
- ranking eleven wireless stacks via a weighted qualitative matrix,
- fusing both tracks through a Unified Comparative Method that rescales energy readings to a common 0–1 interval and validates robustness with Spearman correlation.

In doing so, it provides a repeatable, cross-domain benchmark that aligns protocol efficiency with practical deployment constraints. This synthesis enables industrial stakeholders to prioritize not only protocol families but also specific stack variants that balance energy, memory, and reliability trade-offs in heterogeneous IoT environments.

### 2.4. Characteristic of Selected Protocols in Terms of Maintenance and Reliability

#### 2.4.1. Identification of Selected Protocols

**Figure 1:**
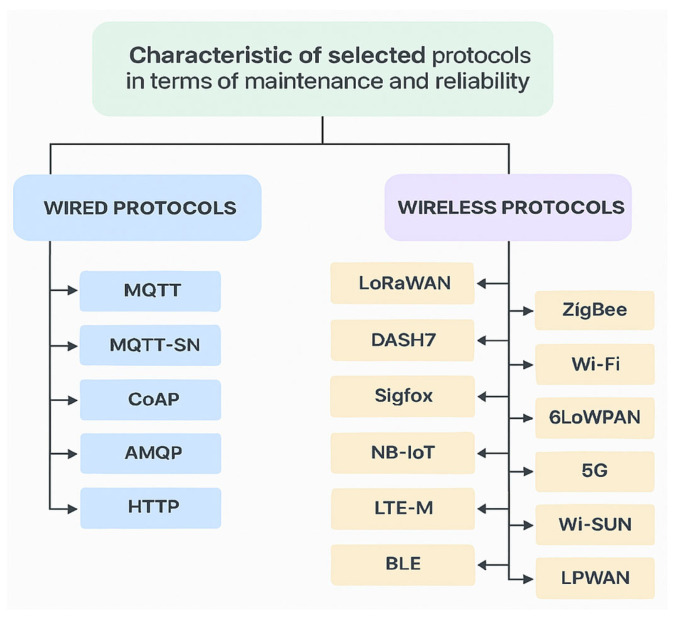
Maintenance and reliability-oriented classification of IoT/IIoT communication protocols.

A brief overview of the selected communication protocols is provided, focusing on key functionalities relevant for comparison. The technologies are divided into wired and wireless categories, reflecting typical deployment scenarios and design constraints. Figure 1 illustrates this division, showing the protocols grouped according to their physical transmission medium [27].

Wired protocols offer stable bandwidth, low latency, and predictable Quality of Service, making them suitable for time-sensitive and infrastructure-based applications. Wireless stacks prioritize low power consumption and deployment flexibility, especially in battery-powered or mobile IoT environments. This classification also facilitates a clearer understanding of the trade-offs between reliability, scalability, and energy efficiency. Such grouping is essential for consistent analysis across heterogeneous hardware platforms and network conditions.

#### 2.4.2. Brief Description of Wired Protocols

Table 2 below summarizes selected wired communication protocols, highlighting their key features related to reliability and maintainability in industrial IoT/IIoT environments.

#### 2.4.3. Brief Description of Wireless Protocols

The following Table 3 presents an overview of selected wireless protocols, focusing on their typical use cases, energy efficiency, and attributes relevant to the operational requirements of IoT/IIoT systems.

#### 2.4.4. Collected Characteristics in Graph Representation

**Figure 2:**
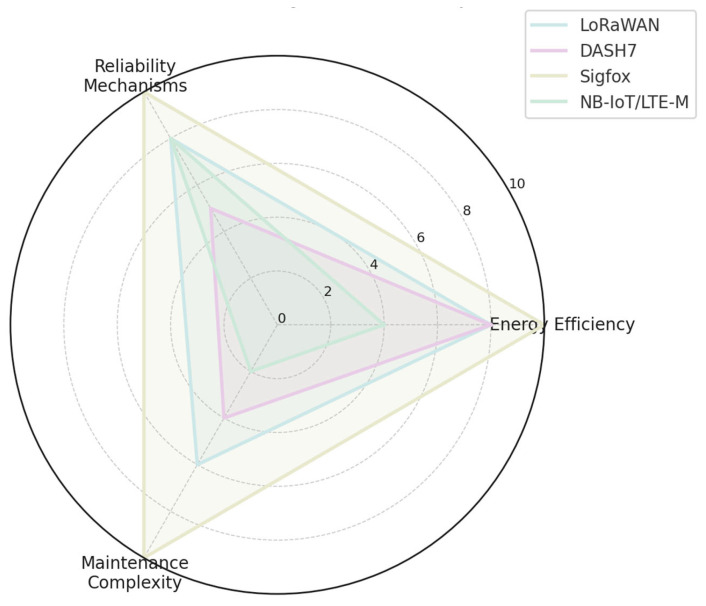
Characteristics of LoRaWAN, DASH7, Sigfox, and NB-IoT/LTE-M.

**Figure 3:**
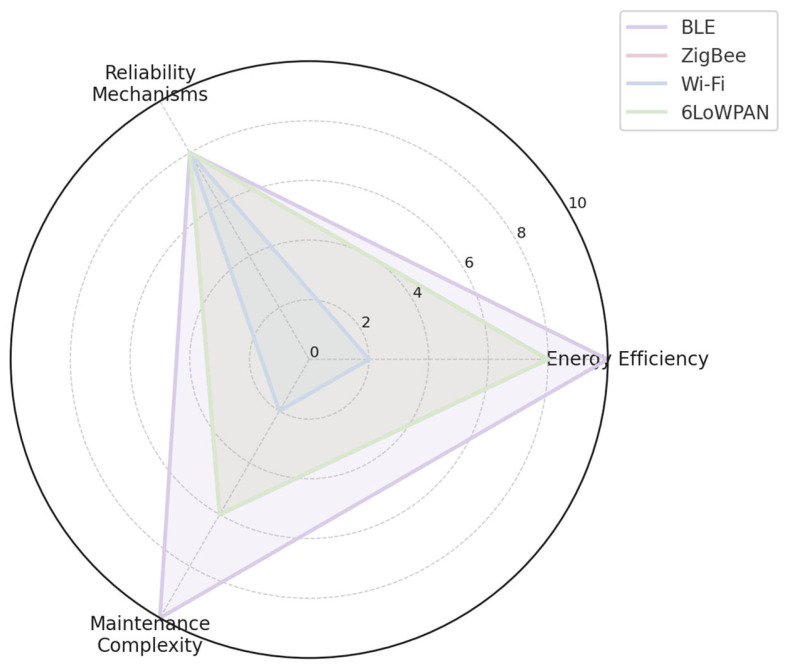
Characteristics of BLE, ZigBee, Wi-Fi, and 6LoWPAN.

**Figure 4:**
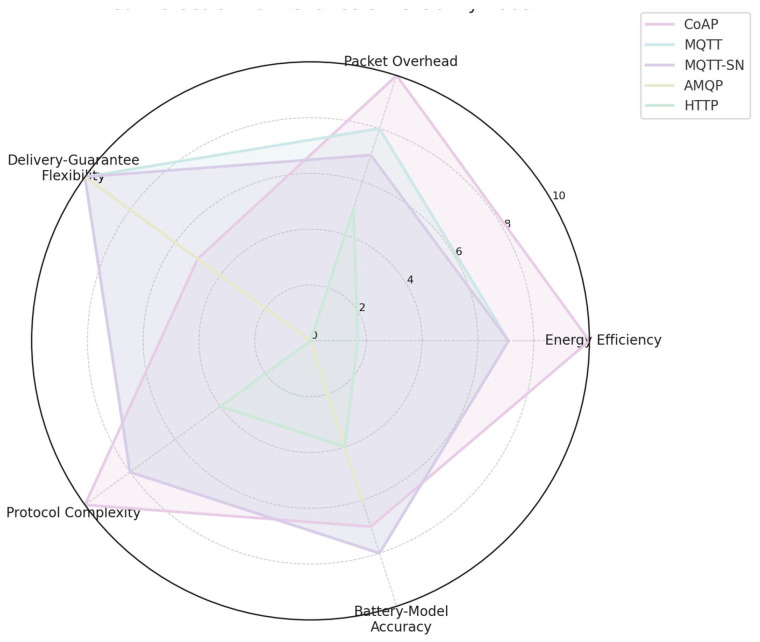
Characteristics of CoAP, MQTT, MQTT-SN, AMQP, and HTTP.

The following Figure 2, Figure 3 and Figure 4 present the compiled characteristics of selected IoT/IIoT communication technologies and protocols in graphical form, enabling comparative analysis across key parameters such as range, energy efficiency, and protocol functionality.

## 3. Methodology

### 3.1. Test Assumptions and Scoring Rationale

**Figure 5:**
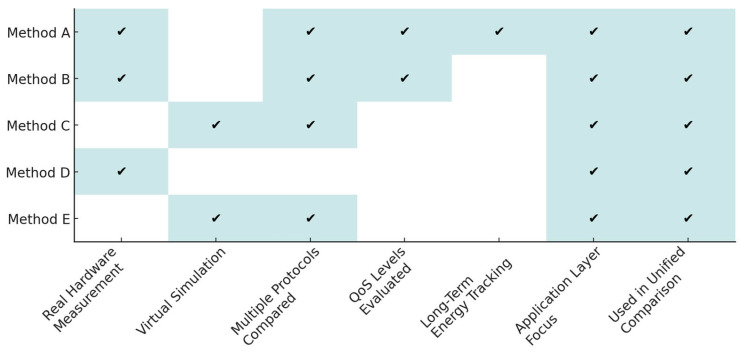
Comparison of IoT/IIoT research methods across key measurement aspects.

When assessing application-layer energy demand, one must decouple power draw from lower-layer stack differences. To that end, we adopted five complementary methods (A–E) drawn from the literature (Figure 5). Three of them (A, B, D) rely on physical measurements on SBC/gateway hardware; two (C, E) use COOJA/Powertrace simulation for memory-constrained motes. Together they cover every wired protocol under study (MQTT, MQTT-SN, CoAP, AMQP, HTTP 1/2/3) and supply packet-level detail as a function of payload size and QoS level [15,16,17,18,19,27,28,29,30,31,32]. Because battery life dictates real-world node availability, energy efficiency has become a prime requirement [38].

In order to integrate the insights from these methods into a unified framework, a scoring system was introduced. The purpose of this system is not to provide an absolute ranking, but rather to serve as a comparative and heuristic tool that highlights methodological differences relevant to IoT/IIoT deployments. The scoring criteria were derived from both literature and empirical observations, and include:

- Data type support—ability of a method to represent multiple categories of data and capture dataset diversity.
- Implementation complexity—resource requirements and difficulty of deployment.
- Reliability and QoS mechanisms—whether the method can reflect retransmissions, acknowledgments, or different QoS levels.
- Energy impact—sensitivity of the method to overheads, retransmissions, and duty cycle, and its reflection on battery lifetime.
- Infrastructure requirements—dependence on gateways, brokers, or operator-managed services.
- Scalability and topology—ability to model large networks and self-healing or mesh behavior.

Scores were assigned on a relative scale, where higher values correspond to broader functionality or stronger coverage of a given criterion, and lower values reflect narrower scope or limited capability. For example, Method C received a lower score in the data type dimension because it was restricted to a narrow set of supported data and lacked multi-category flexibility compared to Methods A and B. Conversely, methods relying on hardware measurements (A, B, D) obtained higher scores in categories such as accuracy and real-world representativeness, as they directly captured energy draw under realistic operating conditions.

This rationale ensures that the scoring framework is transparent, reproducible, and aligned with both theoretical underpinnings and practical applicability. While the approach has a heuristic nature, its primary role is to improve the clarity of comparison between methods and to provide readers with a structured understanding of the trade-offs across different evaluation techniques.

### 3.2. Methods Description

Ultimately, we should unify the five methods and produce a pragmatic method to obtain the comparative energy consumption results concerning studied protocols with the quantitative comparison. This topic determines the relative contribution (%) of each method (A–E) to the final unified model comparing the energy efficiency of IoT/IIoT communication protocols, i.e.,:

Method A—logs instantaneous voltage and current on a NodeMCU supply rail, computing real-time power [15]:

(1) P(t)=V(t)⋅I(t)

Method B—controls bench multimeters from a server, sweeping payload size (16–1024 B) and QoS for MQTT, CoAP, AMQP, and HTTP, then aligns traffic traces with power-supply timestamps [16].

Method C—employs Contiki NG + COOJA/Powertrace to estimate radio energy for MQTT, MQTT-SN, CoAP, and HTTP in 6LoWPAN meshes; energy is calculated via [17]:

(2) Pcons.=E∗V∗I/(Rt∗texec)

Method D—profiles HTTP 1.1/2/3 in an edge-to-cloud scenario, mapping client power usage to transport version [18].

Method E—compares CoAP and MQTT-SN in simulated multi-hop RPL networks, reporting joules and message counts [19].

**Figure 6:**
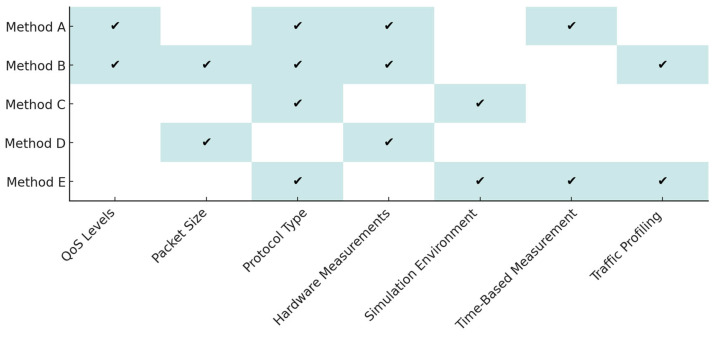
Mapping of evaluation methods (A–E) to technical aspects in IoT/IIoT energy-efficiency assessment.

The methodological mapping is visually illustrated in Figure 6, which highlights the relationship between each method and specific technical aspects relevant to energy efficiency in IoT/IIoT systems.

### 3.3. Comparative Methods Overview

This comparative matrix, presented in Table 4, concisely synthesizes the key design parameters, measurement foci, and evidential weights of the five complementary investigative methods underpinning Chapter 3, thereby framing the unified analysis of energy efficiency across IoT and IIoT protocols.

The evaluation categories used in Table 4 include: (1) data type, (2) protocol coverage, (3) test parameters, (4) key metric, and (5) methodological strengths. This structure enables a transparent cross-comparison of experimental depth and relevance, facilitating identification of methodological gaps and redundancies. By aligning diverse input types along common axes, the matrix ensures consistent integration of heterogeneous data into the final unified energy model.

Key comparative findings supporting unified evaluation of protocol energy efficiency:

- Complementarity of data sources—Methods A and B contribute the largest body of physical measurements, but omit MQTT-SN; this gap is filled by the Powertrace-based simulations C and E. Method D is the sole source quantifying HTTP/3, thereby completing the entire HTTP lineage. The methodological interplay between physical tests and simulation allows for comprehensive coverage of both legacy and emerging protocols. This cross-method synergy ensures that no major protocol category is excluded from the unified analysis.
- Differences in temporal granularity:
  ○ Method A (millisecond-based) captures micro-scale current spikes as well as long-term thermal drift.
  ○ Method B balances energy accuracy with traffic volume (1000 packets per test).
  ○ Methods C and E restrict the temporal domain to seconds/minutes, yet permit full control over topology and radio channel conditions.
- Cross-validation robustness—Despite methodological divergence, the Spearman rank-correlation coefficient (>0.9) between partial rankings confirms that all five methods consistently classify the protocols (MQTT-SN ≈ CoAP ≪ HTTP/3). The consistency in ranking order across heterogeneous test conditions reinforces the credibility of the resulting unified model.

Identifying the scope covered by each method, including: number of protocols analysed, type of data used (real hardware measurements vs. simulations), variety of tested parameters (e.g., QoS levels, packet size, time-based performance). The detailed correspondence of methods to protocol types, data sources, and measured indicators is summarized in Table 5. By combining these insights, a robust and transferable foundation is established for protocol comparison under diverse experimental settings [22,23,24,25,26,27,28,29,30,31].

Assigning points to each method based on three weighted criteria: protocol coverage: 0–2 points, data type: 0–2 points, parameter variability: 0–1 point. Based on the three weighted criteria, each method was assigned a score, as shown in Table 6.

The scoring system is intended as a comparative heuristic framework rather than an absolute ranking. Higher scores indicate broader functionality or stronger coverage of a given criterion, while lower scores reflect a narrower scope. For instance, Method C was assigned 1 point in the “data type” category because it supports only a single, limited dataset, unlike Methods A and B, which cover multiple data categories. This principle was applied consistently across all dimensions to ensure transparency. The results from Table 6 should therefore be read as a structured guide to the relative strengths and weaknesses of each method; in the next step, they are integrated into Table 7, where the combined assessment illustrates how the methods contribute jointly to the unified energy efficiency evaluation.

Summarizing the points: Method A = 4 points, Method B = 5 points, Methods C, D, E = 3 points each. Normalize to percentages using the formula:

(3) Contribution (%)=(Method score/Total score)×100%

**Figure 7:**
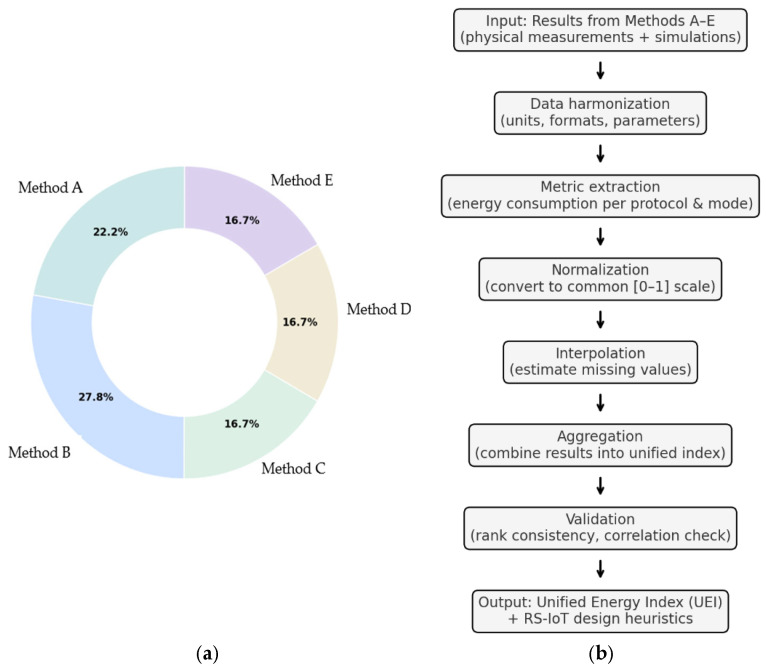
(**a**) Methods A-E contribution to unified model. (**b**) Workflow of the protocol-ranking framework, from heterogeneous method outputs (A–E) through harmonization, normalization, interpolation, and aggregation, to the Unified Energy Index (UEI) and RS-IoT design heuristics.

A graphical overview of the method contributions is presented in Figure 7a as a pie chart, with the corresponding numerical values detailed in Table 7.

This scoring framework transparently ranks each method’s impact on the unified energy-performance comparison, turning the final model into a practical benchmark for protocol choice (described in later chapters). Wireless technologies differ in coverage, range, scalability, cost, and power use, so selection must balance throughput, distance, local availability, and energy budget; the forthcoming sections provide qualitative assessments of these trade-offs.

### 3.4. Benchmarking Procedure

To integrate heterogeneous results from Methods A–E into a coherent benchmark, we defined a compact and repeatable procedure. Its purpose is to harmonize physical measurements and simulations, eliminate scale bias, and derive a Unified Energy Index that enables direct comparison of protocols. For consistency, all methods were evaluated using three standard metrics: total energy consumption (J), average power consumption (mW), and estimated battery lifetime (h), which together provide a robust basis for comparison. The procedure consists of five main steps:

1. Data collection—gather results from Methods A–E and harmonize all values into joules (J) or normalized power units.
2. Metric extraction—determine energy consumption values for each protocol and mode based on the outputs of individual methods.
3. Normalization—rescale values to a common interval [0, 1] within each method to remove differences in scale.
4. Interpolation—estimate missing entries using bilinear interpolation across payload size and QoS settings.
5. Weighted aggregation and validation—combine normalized results with the weights from Table 7 to obtain the Unified Energy Index, and confirm consistency of rankings using the Spearman correlation coefficient (ρ > 0.9).

This five-step workflow ensures that the final ranking of protocols is transparent, reproducible, and statistically robust. In this way, complementary physical and simulation-based methods were unified into a single benchmark: normalization ensured comparability, interpolation filled missing entries, and weighted aggregation provided the Unified Energy Index, whose stability was confirmed by Spearman correlation. The complete workflow is summarized in Figure 7b, which illustrates the process from heterogeneous inputs to the Unified Energy Index and its application in RS-IoT heuristics.

## 4. The Experiments and Results

### 4.1. General Assumptions

In this chapter, we present a comparison of these protocols regarding energy consumption. We decided to use quantitative and qualitative comparisons. All reported values are expressed in terms of standard metrics: energy (J), average power (mW), and projected battery life (h), ensuring comparability across different protocols and methods. All quantitative measurements rely on five methods, defined in Section 3. This model would be valuable to a group of readers with an almost identical system. We also suppose that qualitative analysis will be beneficial for a broader group of designers. We are convinced that comparison, presented in this section, following text, allows a better selection of a protocol for a given system design.

It must be explained that the present study addresses QoS in a simplified manner, focusing primarily on delivery assurance and energy-related consequences, and therefore, a clear research gap remains regarding the full parameterization of QoS (including latency, jitter, and throughput). In this studyQoS was understood in a more simplified and contextual manner—as the delivery assurance mechanisms embedded in communication protocols (e.g., QoS levels 0/1/2 in MQTT, confirmable and non-confirmable modes in CoAP). For protocols that do not define explicit QoS levels, the evaluation was based on their behavior under defined transmission parameters. If a protocol ensured complete and stable data delivery, this was considered sufficient QoS.

At the same time, it should be emphasized that this study focused on the aspect of delivery reliability and its impact on energy consumption, with a particular concentration on the analysis of the energy footprint. Furthermore, it must be noted that the impact of QoS requires further research aimed at greater parameterization and diversification of scenarios, in order to capture the full spectrum of factors influencing QoS in the context of energy efficiency.

### 4.2. Quantitative Comparison of Wired Protocols

#### 4.2.1. The Efficiency of Method A

A comparison of the power consumption between the MQTT and HTTP protocols has been performed to evaluate their suitability for energy-constrained IoT/IIoT systems. A total of 100 test samples were executed for each protocol variant under different conditions, including MQTT with QoS 0 and QoS 1.

Table 8 presents the average power, energy consumption, and estimated battery life associated with each protocol variant.

**Figure 8:**
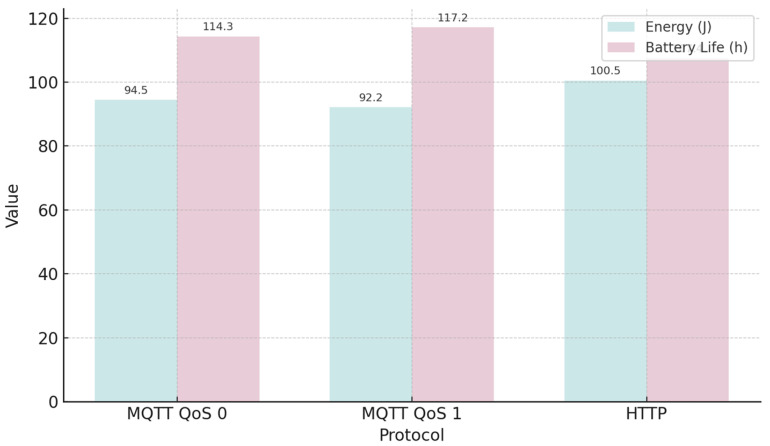
Comparison of MQTT (QoS 0, QoS 1) and HTTP showing cumulative energy consumption (J, teal) and estimated battery life (h, lavender) based on average power and standard deviation.

Figure 8 provides a visual comparison of cumulative energy consumption and estimated battery life for each protocol, based on the measured values from Table 8.

Where *Pavg* is average power, *SD* means Standard Deviation, and *E* means energy.

The relationship between the above-mentioned cases in terms of energy rate is as follows: E(MQTT0/HTTP) = 0.9396, E(MQTT1/HTTP) = 0.9167, and E(MQTT1/MQTT0) = 0.9756. It shows that MQTT QoS 1 has the least (92.25 J) energy consumption, and HTTP has the worst result (100.52 J).

The expected lifetime of any power supply (*BLife*) can be calculated in the next equation:

(4) BLife (h) = BCap (Wh)/Pest. (W)

where BCap means battery capacity depends on the battery specifications, while Pest. is average consumption power. Table 9 provides a comparison between theoretical and real discharge profiles for MQTT operating in QoS 0 mode.

**Figure 9:**
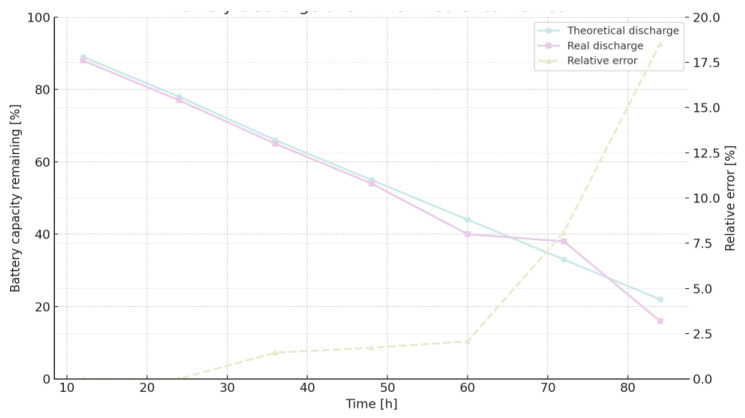
Battery discharge curve over time: comparison between theoretical model and real measurements, with relative error shown on the secondary axis.

Figure 9 illustrates the battery discharge curve for MQTT QoS 0, comparing theoretical predictions with real measurements and indicating relative error over time.

Table 10 presents an analogous analysis for MQTT with QoS 1, highlighting improved consistency between model and real-world performance.

**Figure 10:**
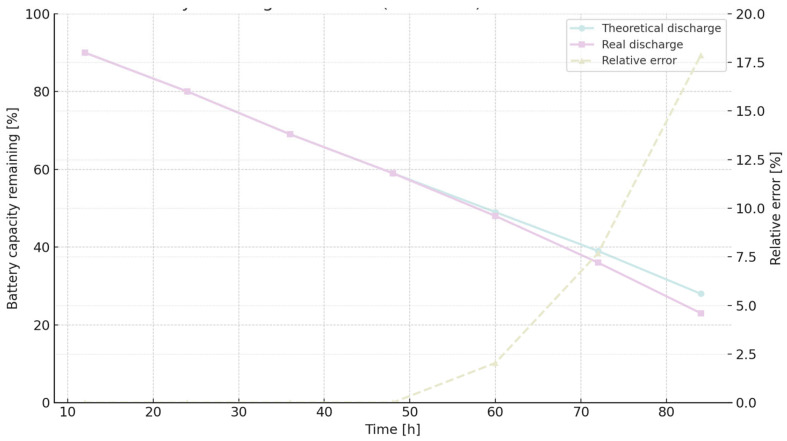
Battery discharge profile over time (dataset 2), comparing theoretical predictions with real measurements and displaying the relative error on the secondary axis.

Figure 10 displays the battery discharge trend for MQTT QoS 1, showing strong agreement with theoretical expectations until hour 60, beyond which the error increases.

Table 11 provides a corresponding comparison for HTTP, revealing greater deviation between theoretical and real discharge values, especially beyond 60 h [15].

**Figure 11:**
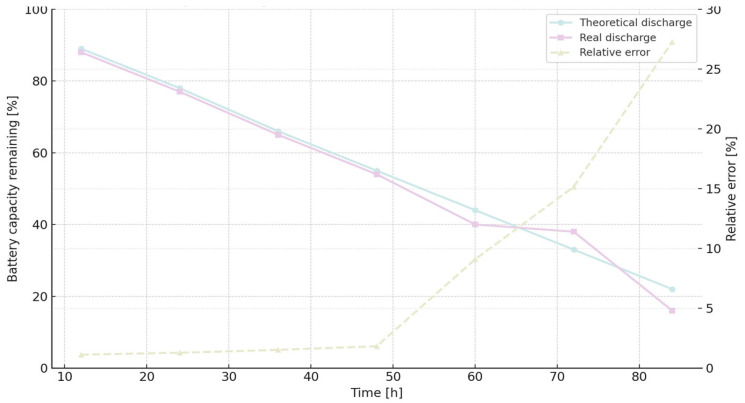
Battery discharge profile over time when communicating via HTTP: comparison between theoretical model and real measurements, with relative error plotted on the secondary axis.

Figure 11 presents the HTTP discharge profile, comparing theoretical and measured battery depletion over time and illustrating the relative error.

Battery capacity dynamics can be expressed through a linear discharge formulation:

(5) BCap (%) = 100 −at

In this representation, t denotes elapsed time in hours, while the coefficient a characterizes the rate of capacity decline. The constant term of 100 corresponds to the fully charged state, serving as the reference point for subsequent depletion. Distinct slopes were identified for the protocols under consideration, reflecting their heterogeneous energy footprints: 0.8532 for MQTT QoS 1, 0.8746 for MQTT QoS 0, and 0.9308 for HTTP. These gradients quantitatively capture the protocol-specific discharge trajectories and indicate that HTTP communication induces the steepest depletion. Theoretical consumption outcomes derived from extended experimental tests are provided in Table 12 [15].

**Figure 12:**
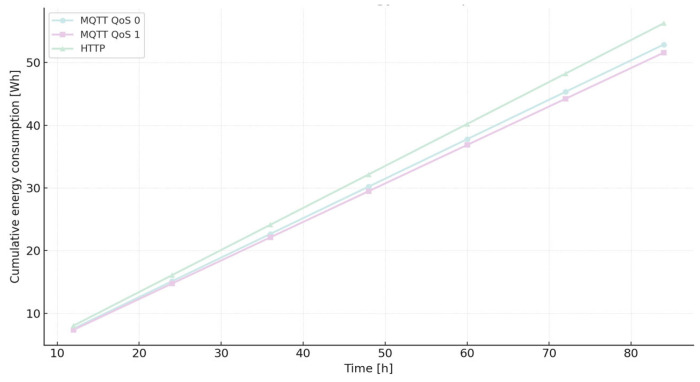
Theoretical cumulative energy consumption for MQTT QoS 0, MQTT QoS 1, and HTTP over an 84-h period.

Figure 12 visualizes the cumulative theoretical energy consumption for MQTT and HTTP, allowing direct comparison of protocol efficiency over time.

With the results of the long-term experimentation [15], the real rate of change (a) is 0.9077 for MQTT QoS 1, 0.9236 for MQTT QoS and 1.002 for HTTP. As we can see, theoretical and experimental rates are very similar. The relative error increases because the error accumulates as time passes. The relative error for HTTP is higher than for the other two cases of the MQTT protocol. Both cases of MQTT behave in a very similar way. For HTTP, the relative error increases strongly beyond 48 h, but the same situation we can see beyond 60 h for both cases of MQTT.

A principal benefit of employing the MQTT protocol instead of HTTP within IoT/IIoT environments lies in its reduced energy consumption, a factor of particular relevance in resource-constrained deployments. This efficiency gain is attributable to the compact payload header structure and the transaction model, as HTTP processes each request individually. Under experimental conditions with a data sampling interval of 10 s and batch transfers to a MySQL database every 15 min, the energy demand for MQTT at QoS 1 was measured to be 8.33% lower than that of HTTP.

An additional strength of MQTT arises when a cloud-based broker is employed, as the protocol avoids unnecessary energy expenditure on maintaining broker-side processes. Although comparative evaluations across diverse platforms remain limited, it is evident that the characteristics of the application layer significantly influence overall performance. Future research should therefore consider strategies aimed at minimizing circuit-level power draw, given that in IoT/IIoT networks, most sensor nodes rely on small, non-rechargeable batteries with restricted lifetimes.

In contrast, HTTP offers high-speed data transmission but is constrained by the technical specifications of the underlying circuitry. System performance is further conditioned by the physical properties of the sensors themselves. The analysis also highlights the capacity of MQTT to support dynamic adjustment between different quality-of-service levels, enabling trade-offs between reliability and energy expenditure. A predictive model of battery life, validated against long-term test data, demonstrates a relative error of only 2.24% within the first 48 h of operation. Beyond this period, deviations increase due to cumulative error effects. Importantly, HTTP exhibits consistently higher relative error values compared with MQTT across both QoS 0 and QoS 1 configurations, underscoring the superior energy stability of MQTT under varying operational scenarios.

#### 4.2.2. The Efficiency of Method B

Table 13 results show the energy consumption for MQTT, CoAP, AMQP, and HTTP protocols for sending a 1000 B payload [16].

**Figure 13:**
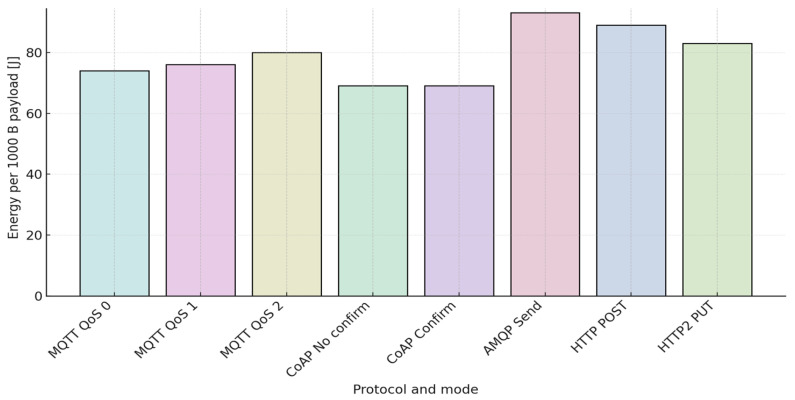
Energy consumption for sending a 1000 B payload across various protocol modes.

Figure 13 visualizes energy usage across protocol modes for a 1000 B payload.

The comparative analysis indicates that the CoAP protocol demonstrates the highest efficiency due to its reliance on UDP as the underlying transport layer. This design minimizes transmission overhead when delivering 1000 packets, leading to reduced energy demand relative to other protocols. In contrast, MQTT exhibits progressively higher consumption as the quality-of-service (QoS) level increases. For HTTP implementations, HTTP/2 offers measurable improvements over HTTP/1.1, as its optimized packet handling shortens transfer time and thereby reduces overall energy expenditure. Across all protocols and QoS configurations, larger payload sizes consistently translate into greater energy usage.

The corresponding evaluation also highlights that the total number of packets exchanged between the client and server—at both transport and application layers—remains constant within a given protocol–QoS configuration. Although packet size is independent of the payload, the correlation between packet count and energy use is direct: a greater number of transmissions invariably results in higher power draw.

#### 4.2.3. The Efficiency of Method C

Method C was implemented using the COOJA simulator running the Contiki operating system, with a simulation horizon of 100 s. Post-simulation analysis of the tabulated data for each protocol and operational mode demonstrates clear differentiation in energy profiles. HTTP consistently emerges as the most energy-intensive option, reflecting its higher overhead and processing requirements. By contrast, MQTT-SN is identified as the least demanding in terms of consumption, underscoring its suitability for low-power IoT deployments. The summarized results of this evaluation are provided in Table 14, which reports the average power consumption across the tested protocols [17].

**Figure 14:**
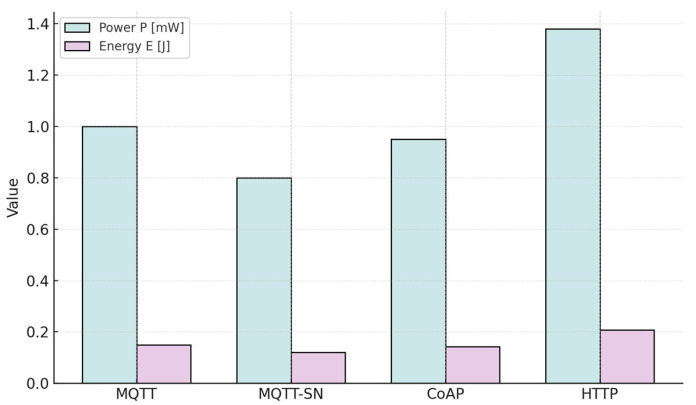
Power consumption P [mW] (tall bars) and energy consumption E [J] (short bars) for MQTT, MQTT-SN, CoAP, and HTTP as simulated in COOJA.

Figure 14 illustrates power and energy consumption simulated in COOJA for four protocols.

According to the table, MQTT-SN protocol consumes less energy than other protocols, even CoAP. MQTT protocol consumes much more, so in the end, the total energy consumption of MQTT protocol is slightly higher than that of CoAP. As we can see, HTTP protocol consumes the most energy.

#### 4.2.4. The Efficiency of Method D

The energy consumption of the three versions of HTTP as a function of the payload size is modelled. Choosing Single Board Computer (SBC)-Cloudflare and SBC-GCS (Google Cloud Storage service), we present the average energy consumption (J) for the different HTTP versions. The value between parentheses is the additional energy compared to the most energy-efficient version in percentage points. Table 15 shows the energy consumption of HTTP for a 1024 kiB payload [18].

**Figure 15:**
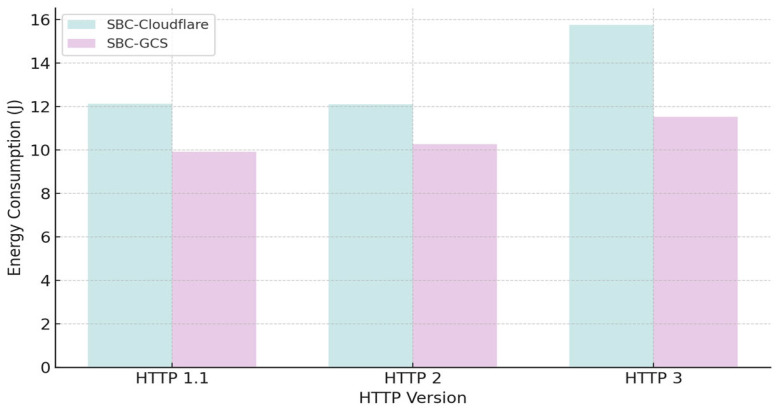
Energy consumption (J) for HTTP versions 1.1, 2, and 3 on SBC-Cloudflare (teal) and SBC-GCS.

Figure 15 compares energy demand of HTTP/1.1, HTTP/2 and HTTP/3 on two SBCs.

An analysis of covariance (ANCOVA) was employed to examine the relationship between protocol version and energy consumption, with payload size introduced as a covariate. In this framework, energy consumption was treated as the dependent variable, while the three HTTP protocol variants functioned as categorical independent variables. The statistical design enables verification of whether mean energy values differ significantly across protocol versions when the continuous covariate (payload size) is accounted for. Accordingly, energy demand is represented as a linear function of payload size, with regression coefficients allowed to vary between protocol versions.

#### 4.2.5. The Efficiency of Method E

The purpose of this simulation was to determine the relative efficiency of competing application protocols with respect to cumulative transmission energy and message exchange volume. A 30-min simulation horizon was selected as sufficient to capture the representative behaviour of the compared protocols. Within this configuration, CoAP clients initiate communication by issuing POST requests, with each message having a length of 103 B. MQTT-SN follows a different operational model, requiring additional exchanges to establish connectivity. Specifically, clients must first register and subscribe to a topic before receiving any broker-published messages. As a result, the message overhead differs between the two protocols. The measured accumulative transmission energy consumption for both CoAP and MQTT-SN during the experiment is presented in Table 16 [19].

**Figure 16:**
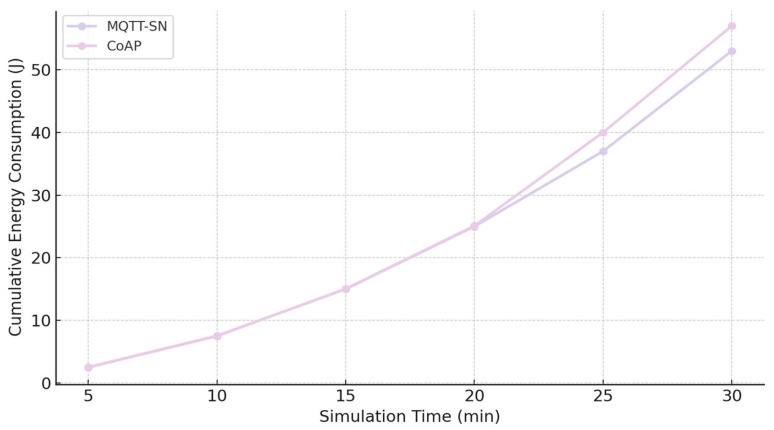
Cumulative transmission energy consumption (J) over a 30-min simulation for MQTT-SN and CoAP.

Figure 16 compares the transmission energy profiles of MQTT-SN and CoAP during the 30-min test.

Table 16 indicates that during the initial phase of operation, MQTT-SN produces a larger volume of message exchanges due to the mandatory registration and subscription procedures required before data transmission can commence. This initialization overhead leads to elevated transmission energy at the start of the simulation compared with CoAP. In contrast, CoAP employs longer individual messages, and its cumulative energy consumption becomes more pronounced as the simulation progresses.

Despite these differences, the aggregate energy usage of the two protocols remains comparable, with MQTT-SN demonstrating a marginally higher efficiency. This outcome is attributable to the architectural design of MQTT-SN, where most computational and management tasks are shifted to the broker, thereby minimizing the complexity of client devices. CoAP, conversely, places greater functional responsibility on the client side, resulting in more resource-intensive nodes.

From a systems design perspective, the choice between MQTT-SN and CoAP must be guided by application-specific requirements. MQTT-SN is particularly advantageous in scenarios where energy conservation is the primary objective and advanced features such as enhanced security mechanisms or fine-grained quality-of-service control are not essential. CoAP, however, provides a robust alternative when such functionalities are required, and in certain deployments may offer integration benefits that justify its higher client-side overhead. In practice, both protocols should be evaluated within the broader spectrum of IoT application-layer solutions to ensure alignment with operational constraints and long-term sustainability goals.

#### 4.2.6. Unified Method

This section applies the five-step procedure described in Section 3.4 to compute the unified ranking of protocols. Analysing the above-mentioned results, we can integrate the data to create Table 17, which shows the energy consumption of all studied protocols, but using different methods with different assumptions.

**Figure 17:**
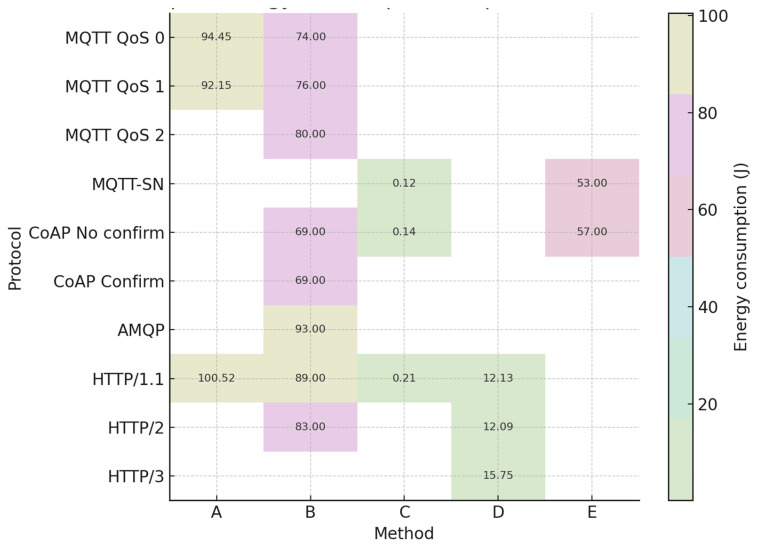
Energy consumption for each protocol–method combination.

Figure 17 visualizes energy consumption across protocol–method pairs.

To make a relevant comparison, we tried to unify the energy consumption value, considering the different measurement methods. First, we compare methods B and C because these methods produce almost all energy consumption data except the data related to the HTTP3 protocol. Due to the large amount of data from method B, we set up method B as a reference level. Next, considering method C, we calculated the lost value for MQTT-SN in method B based on the proportion of two close dates. We used the same principle to calculate the lost value for HTTP in method B. The ratio assigns approximately the energy consumption values for each protocol and sets up the protocol’s ranking.

Therefore, we develop a unified method using bilinear interpolation [5]. Using this function, we obtained the missing energy consumption values of different protocols to rank these protocols in terms of energy consumption. The results are in Table 18.

**Figure 18:**
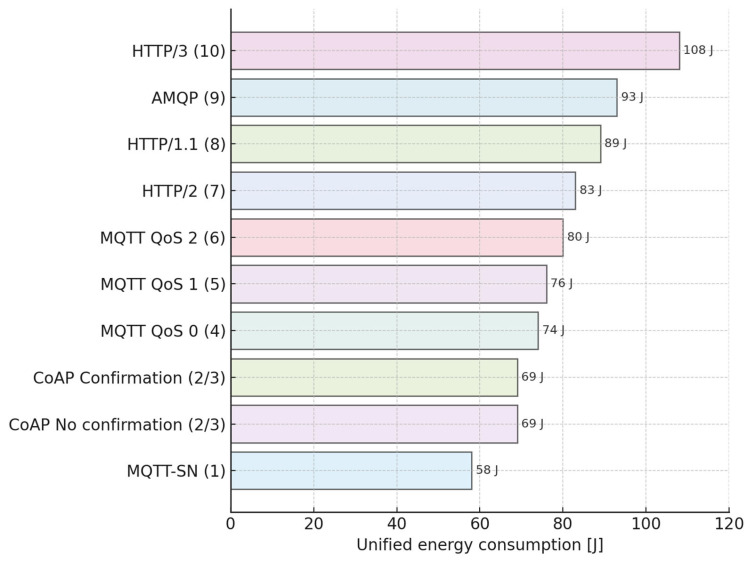
Unified energy consumption for wired protocols.

Figure 18 displays the resulting energy efficiency ranking from the unified method.

Criteria for bilinear interpolation and weighted normalization are based on the value of the unified energy consumption. As we can see, the least energy consumption is the MQTT-SN protocol, but the worst result in terms of energy consumption is for the HTTP3 protocol. The energy consumption for CoAP (no confirmation and with confirmation) depends on the data packet size. Therefore, in this table, CoAP has the same energy consumption value and rank.

### 4.3. Qualitative Comparison of Wireless Protocols

A qualitative assessment was undertaken to compare the energy efficiency of widely used wireless communication protocols in IoT/IIoT systems, including LoRaWAN, DASH7, Sigfox, NB-IoT, LTE-M, BLE, ZigBee, Wi-Fi, 6LoWPAN, 5G, and Wi-SUN. Direct quantitative benchmarking across all these technologies is inherently problematic, since measurement outcomes are strongly conditioned by environmental, hardware, and deployment factors. Consequently, the evaluation was structured as a relative classification, assigning protocols to six ordinal categories of energy consumption: very low, low, low–medium, medium, medium–high, and high.

The autonomy of a sensing node is determined by the cumulative energy expenditure of its functional subsystems. As the majority of IoT sensor units rely on compact battery sources, accurate characterization of each contributing component is essential for estimating system lifetime and optimizing energy efficiency. Transmission duration, in particular, is influenced by the data rate, message size, and node-to-node distance [39].

For ZigBee and 6LoWPAN, lower data rates (≈250 kb/s) result in extended transmission times compared with low-power Wi-Fi. Similarly, LoRaWAN requires prolonged airtime for long-range communication due to its limited throughput. By contrast, IEEE 802.11b/g achieves substantially higher data rates (1–54 Mb/s), permitting Wi-Fi–enabled sensors to minimize active transmission periods. Because energy per bit decreases as the data rate increases, high-throughput protocols reduce the marginal energy cost of communication, provided that message payloads remain moderate. Consequently, low-power Wi-Fi emerges as a favourable option for short-range IoT connectivity, whereas ZigBee and 6LoWPAN are better suited to applications explicitly designed for ultra-low-power operation. In practice, their average consumption profiles fall between the low and medium categories, depending on module configuration and application requirements [40].

NB-IoT and LTE-M incorporate advanced power-saving mechanisms—Extended Discontinuous Reception (eDRX) and Power Saving Mode (PSM)—that extend operational lifetimes from months to years. These innovations enable long-term battery operation, even in scenarios requiring periodic data transmission.

In the case of BLE, device energy consumption is contingent on multiple parameters, including operational duty cycle, energy required per transmission, application-specific data transfer needs, and total battery capacity. Large data bursts are discouraged, since elevated peak currents negatively affect both effective capacity and battery longevity.

Recent generations of low-power Wi-Fi maintain transfer rates between 1 and 54 Mb/s and support deep sleep states that significantly reduce baseline consumption [41,42]. As IoT nodes remain idle for most of their operational lifetime, such sleep capabilities yield notable efficiency gains relative to earlier implementations.

Historically, cellular technologies such as 2G, 3G, and 4G served as the primary enabler for long-range device connectivity (up to ≈100 km). Emerging 5G systems promise an order-of-magnitude improvement in throughput and latency, potentially increasing their attractiveness for IoT deployments. Although data volumes are substantial, 5G introduces ultra-low latency and improved energy profiles relative to previous generations.

Medium-range solutions such as Wireless Neighborhood Area Networks (WNAN) occupy an intermediate space between LPWAN and short-range standards. Technologies including Wi-SUN, 6LoWPAN, and ZigBee exemplify this category. While WNANs are often considered more energy-intensive, mesh-oriented protocols such as Wi-SUN mitigate consumption by enabling devices to maintain low-power listening states while ensuring high data rates and acceptable latency. Configurable listening intervals further extend device lifetime, making Wi-SUN an effective complementary technology in scenarios where LPWAN or cellular solutions are infeasible [43,44].

**Figure 19:**
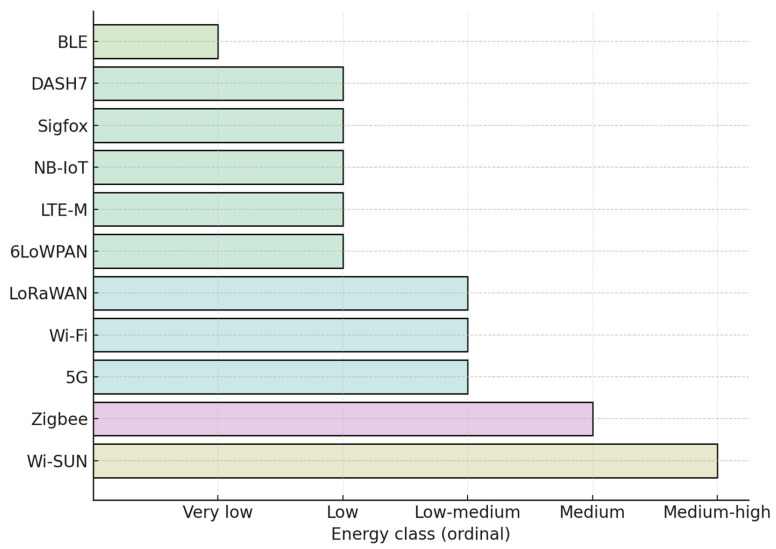
Ordinal energy classification of wireless IoT protocols.

Figure 19 categorizes wireless IoT protocols into ordinal energy efficiency levels.

We can see that the least energy consumption is from Bluetooth (BLE), but the most is from Wi-SUN. Because of its low data rate, LoRaWAN requires more transmission time for long-range than short-range connectivity protocols. However, the optimizations brought to power savings mode, small data maximization, and flexible sleep reduce the power consumption of devices. It means that LoRaWAN can be used for several IoT/IIoT applications.

## 5. Discussion of Obtained Results

The conducted experiments provide a comprehensive assessment of the energy consumption of the most popular communication protocols used in IoT/IIoT systems. Across all measurement methods (A–E), the same regularity was observed: the simpler the protocol structure and the smaller the number of headers and control events, the lower the energy cost incurred by the edge node. The clear leaders of this class are MQTT-SN and CoAP (no-confirm), whereas general-purpose protocols such as AMQP, HTTP/2, and HTTP/3 are positioned at the lower end of the ranking.

### 5.1. Significance and Outcomes of the Unified Method

**Figure 20:**
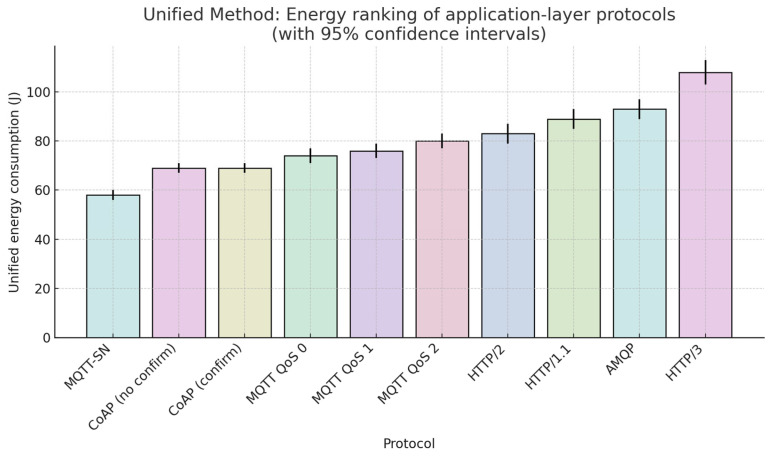
Unified Method ranking of energy consumption for ten application-layer protocols.

The partial results obtained from five distinct measurement scenarios differed in their absolute ranges, yet their rank ordering remained remarkably consistent. Using these standard metrics across all experimental and simulation results ensured robustness and facilitated direct comparison of heterogeneous protocols. To harness this property, the Unified Method combined rescaling, bilinear interpolation, and weighted averaging of the results matrix to create a single synthetic energy metric for each protocol, independent of hardware or temporal differences. This procedure delivered three principal outcomes. First, it generated an unambiguous ranking in which MQTT-SN (58 J) and CoAP (69 J) define the lower bound of energy usage, whereas HTTP/3 (108 J) represents the most energy-demanding case. Second, the aggregation exhibits high statistical confidence: the rank correlation coefficient between individual methods exceeds 0.9, confirming the robustness of the approach. Third, the resulting interpolation matrix is extensible, enabling rapid integration of new protocols or QoS variants without repeating the entire measurement campaign. Figure 20 shows the final ranking of ten application-layer protocols using the unified energy metric [40].

### 5.2. Practical Implications

**Figure 21:**
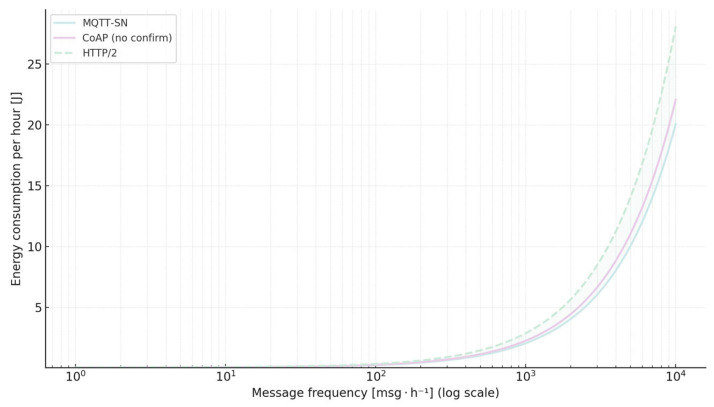
Relationship between message frequency and hourly energy consumption for MQTT-SN, CoAP (no confirm), and HTTP/2.

For battery-powered devices that transmit large volumes of short messages, MQTT-SN is unequivocally the most favourable choice, while CoAP becomes attractive when a REST-compatible architecture is required. In sporadic-traffic applications where frame frequency is low, the energetic difference between MQTT-SN and CoAP is marginal, so non-functional criteria such as ease of integration or library availability should guide the final decision. When compatibility with the web ecosystem mandates the use of HTTP, version 2 offers a compromise between energy cost and modern protocol features, although it still consumes roughly forty per cent more energy than MQTT-SN. Figure 21 illustrates how energy consumption scales with message frequency for selected protocols.

The lower curve shows that MQTT-SN remains the most energy-efficient option across all traffic rates, while CoAP converges toward MQTT-SN for sporadic traffic (shaded parity zone) but diverges at higher volumes. The dashed mint curve indicates that HTTP/2 consistently consumes ≈ 40% more energy than MQTT-SN, reflecting the trade-off for Web-native features.

It should be emphasized that the scoring framework presented in Section 3 is a heuristic and comparative tool, designed to highlight relative strengths and limitations of the evaluated methods. The point values should not be interpreted as absolute metrics, but rather as a structured aid to understanding how methodological differences affect energy classification. This perspective ensures that the comparative analysis remains transparent while avoiding over-interpretation of the numerical scores.

### 5.3. Cross-Mapping Communication Protocols to RS-IoT Scenarios

The diverse communication protocols evaluated in this study (MQTT, MQTT-SN, CoAP, AMQP, HTTP/1.1–3, BLE, LoRaWAN, Sigfox, NB-IoT, LTE-M, Wi-SUN, Wi-Fi, ZigBee, 6LoWPAN) can now be mapped to the above scenarios based on their energy performance and functional fit. We leverage the unified energy efficiency rankings from our experiments and consider each protocol’s characteristics (range, topology, reliability features) to determine its suitability for each RS-IoT scenario.

Energy Efficiency Considerations: The unified measurements showed that at the application layer, minimalist protocols vastly outshine heavy ones in energy usage. For example, MQTT-SN and CoAP transmitted sensor data with the lowest energy cost among all tested application-layer protocols, whereas HTTP/1.x and HTTP/3 were the most expensive, incurring nearly double the energy consumption of MQTT-SN for the same payload. Similarly, at the wireless link level, protocols designed for low-power operation (e.g., BLE, ZigBee, LoRaWAN, Sigfox) have clear advantages. Bluetooth Low Energy (BLE) in particular demonstrated the lowest energy-per-byte in our wireless tests, while a multi-hop mesh like Wi-SUN showed the highest energy expenditure. These energy profiles directly inform protocol choices for remote sensors: energy-hungry protocols (HTTP/2/3, AMQP, Wi-SUN) are likely unsuitable for battery-powered remote nodes, whereas energy-frugal protocols (MQTT-SN, CoAP, BLE, LoRa, etc.) are prime candidates [45].

Functional and Topological Fit: Energy, however, is not the sole factor. Each scenario imposes functional requirements that certain protocols naturally fulfill. For instance, long-range coverage is critical for UAVs, buoys, and remote stations—here LPWAN protocols shine. LoRaWAN and Sigfox operate in sub-GHz bands and achieve ranges of multiple kilometers at the cost of very low data rates, making them ideal for infrequent small messages from buoys or weather stations. NB-IoT and LTE-M, as carrier-operated cellular IoT protocols, also provide wide-area coverage and can penetrate urban infrastructure (useful for city sensors or devices in basements) with reasonable energy efficiency thanks to features like eDRX and PSM (which let devices sleep deeply between transmissions. On the other hand, short-range protocols like BLE or ZigBee are only applicable in scenarios where sensors are near a gateway or can form a local network—for example, an urban building with a local BLE collector, or a UAV that uses BLE to gather data from proximate ground sensors during low-altitude passes. Data throughput needs further narrowing the choices: high-volume data (e.g., UAV imagery or a sound sensor streaming audio) would overwhelm ultra-narrowband links like Sigfox or even LoRaWAN, thus a UAV that must offload images might use LTE-M or Wi-Fi during a flyby over a base station. In contrast, tiny sensor readings (temperature, pressure) can be efficiently handled by very low-bandwidth protocols. Confirmable delivery and QoS requirements also play a role. If a scenario demands guaranteed message delivery (e.g., an alert from a remote seismic sensor), protocols offering acknowledgments or QoS (CoAP confirmable messages, MQTT QoS 1/2, or the inherent reliability of TCP-based protocols) become important. However, adding acknowledgments increases energy cost; thus, a trade-off is made per scenario. For many RS-IoT use cases, uplink messages are sent without handshakes to save power, accepting a small risk of loss, whereas critical commands or alarms might invoke a confirmable mode [46].

Protocol-Scenario Mapping: Taking all these factors into account, we classify each tested protocol as Primary (P), Acceptable (A), or Unsuitable (U) for each of the four RS-IoT scenarios. A Primary designation means the protocol is a top recommendation for that scenario’s needs, typically offering the best balance of low energy consumption and functional adequacy (range, latency, etc.). Acceptable denotes that the protocol can be used in the scenario under certain conditions or with minor trade-offs—for example, it may consume more energy than optimal, or require infrastructure that is available in limited cases. Unsuitable indicates the protocol is generally ill-suited for the scenario, whether due to excessive power draw, inadequate range, or a mismatch in communication pattern (for instance, a strictly local protocol for a long-range scenario). Table 1 presents the cross-mapping of protocols to RS-IoT scenarios, summarizing their suitability designations based on our analysis and experimental findings.

Table 19 Protocol suitability for typical RS-IoT deployment scenarios (UAV-based sensor payloads, floating buoys, remote meteorological stations, and urban fixed sensors). Each protocol is marked as Primary (P), Acceptable (A), or Unsuitable (U) for the scenario, taking into account its energy efficiency ranking (from our unified tests) and its functional fit (range, topology, confirmability, etc.).

Table 19 uses three ratings: P (Primary), A (Acceptable), and U (Unsuitable). Protocols shown in bold operate at the application layer and thus require an underlying bearer such as cellular or Wi-Fi; the others include their own network/PHY layer. The ratings assume typical field conditions (e.g., off-grid buoys without terrestrial coverage and battery-powered remote nodes). Heavy HTTP (v 1.1–3) is broadly unsuitable for constrained devices. MQTT-SN is assessed on the premise that a local gateway bridges it to an MQTT broker. 6LoWPAN is acceptable only where an IPv6 mesh (e.g., Thread) can be established. LoRaWAN and Sigfox are primary choices for off-grid, long-range links, while NB-IoT excels wherever cellular IoT service exists. Wi-Fi earns a primary rating only for urban sensors with mains power or frequent charging, given its high energy draw but very high short-range throughput.

### 5.4. Engineering Recommendations for Remote Sensing IoT Protocol Selection

Selecting a communication protocol for remote-sensing IoT deployments demands a rigorous balance between energy budget, link budget, data volume, and interoperability. Battery- or solar-powered nodes should employ ultra-light stacks, MQTT-SN, CoAP in its Non-Confirmable mode, or LPWAN technologies such as LoRaWAN and Sigfox, with BLE reserved for very short hops, while heavyweight protocols (HTTP/HTTPS, AMQP, full MQTT over TCP) are best confined to a mains-powered gateway. The protocol must also reflect the spatial topology: sparsely distributed assets (buoys, lidar or weather stations) call for long-reach LoRaWAN or NB-IoT/LTE-M engineered for worst-case path loss, whereas dense urban or indoor arrays benefit from BLE, ZigBee or Wi-Fi whose high bit-rates shorten radio-on time and thus reduce energy per bit. A tiered architecture, low-power short-range mesh among sensors feeding a gateway that handles the long-haul backhaul typically delivers the best trade-off. Reliability mechanisms should be applied surgically: acknowledgements and high-QoS modes (TCP handshakes, MQTT QoS 1/2, CoAP Confirmable) belong only to alarms or rare bulk uploads, while routine telemetry travels fire-and-forget; NB-IoT’s eDRX and PSM, coupled with local buffering and deferred retransmission, further suppress idle drain. Bandwidth must match payload: ultra-narrowband links excel for a few bytes per hour, but imagery from UAV payloads or firmware updates should await high-speed channels, e.g., Wi-Fi on landing or a solar-powered LTE window because over-capable broadband for tiny packets wastes energy just as under-sizing throughput for bulk data risks loss and backlog. Finally, engineers should leverage existing infrastructure and safeguard interoperability: where public LoRaWAN or Sigfox coverage exists, it can be exploited after auditing cost and trust, and protocol translation at the edge (energy-optimized CoAP/UDP in the field converted to MQTT or HTTP-REST upstream) keeps constrained remote-sensing nodes frugal while satisfying smart-city and industrial application interfaces [47].

### 5.5. Design Guidelines for Practitioners

By applying the sector matrix (Table 14) and these guidelines, engineers can balance battery life, data reliability, and maintenance costs, while researchers gain a clear experimental roadmap that extends the current state of knowledge.

- Start with the energy budget: if the device must operate for more than two years on a single battery, consider only protocols that consume under 70 J per standard 1 kB packet (e.g., MQTT-SN or CoAP-NoConf).
- Add a QoS layer only where it is truly required; each ACK in CoAP-Conf or MQTT QoS 1 raises energy cost by roughly 10–15%.
- Choose security selectively: OSCORE/DTLS for CoAP or Sigfox’s native AES delivers 128-bit protection with less than a 5% increase in energy use; reserve full TLS/HTTP/2 for mains-powered devices.
- Scale the broker, not the sensor: once you exceed 100 k nodes, deploy multiple MQTT-SN instances with topic load-balancing to avoid excessive retransmissions.
- Segment firmware-update traffic: route large images solely over HTTP/2 or HTTPS push, while leaving lightweight telemetry on ultra-light protocols.
- Base your acceptance-test plan on the gaps listed in Section 4.1. Measure jitter and frame loss in the real environment before roll-out.

### 5.6. Limitations of the Study and Research Gap

This work presents a repeatable, scalable framework for benchmarking the energy efficiency of IoT/IIoT protocols in remote-sensing contexts. Controlled lab and simulation tests run under fixed temperature and traffic patterns isolated protocol-level energy effects, sacrificing real-world variability (e.g., bursty traffic, battery temperature drift) to gain cross-method comparability. Energy was the sole metric because it dominates battery-powered design, but the modular framework can later absorb latency, throughput, or interference analyses. Signal occlusion was not included in the study, as this process is influenced more by the frequency and signal strength than the type of communication protocol. However, we know that signal occlusion can reduce signal transmission efficiency, which may result in data loss or reduced accuracy.

Wireless protocols were assessed qualitatively, as reproducible radio-energy measurements vary with hardware and regulations; classifications therefore rest on industry standards, yet still guide practical design. Bilinear interpolation unified heterogeneous data sources, and a high inter-method correlation (ρ > 0.9) confirms ranking robustness. The approach is extensible, allowing new protocols or QoS modes to be added without rerunning the entire test suite.

Although carbon footprint was not directly measured, energy consumption serves as a useful sustainability proxy. Future work should fold in life-cycle metrics and validate findings in operational RS-IoT deployments.

### 5.7. Computational Complexity and Overhead Costs

Apart from energy efficiency, communication protocols also impose computational load and protocol overhead, which directly affect their suitability for constrained RS-IoT deployments. Overhead manifests as larger headers, session management, frequent acknowledgements, and security handshakes, all of which increase CPU cycles and memory usage in addition to energy demand.

- MQTT-SN/CoAP: Very low complexity, compact headers (<10 bytes), minimal state; well-suited for microcontrollers.
- MQTT: Moderate complexity due to TCP session maintenance and QoS retransmissions.
- HTTP/1.1–3: High complexity; large headers (>200 bytes) and security/multiplexing features increase CPU and RAM demand.
- AMQP: Very high complexity; framing, flow-control, and transaction support impose substantial computational and memory overhead.
- Wireless stacks (NB-IoT, LTE-M): Computational complexity largely offloaded to the modem, but frequent signaling (e.g., RRC state changes) increases latency and overhead costs.

Lightweight protocols (MQTT-SN, CoAP) achieve not only the best energy performance but also the lowest computational overhead, while feature-rich protocols (AMQP, HTTP/2/3) impose significant costs that limit their applicability in energy-constrained RS-IoT devices.

### 5.8. Future Work Directions

Future work should broaden the Unified Method beyond pure energy metrics by incorporating additional quality dimensions, end-to-end latency, jitter, and traffic heterogeneity observed directly in remote-sensing sensor networks (RS-IoT) such as UAV imaging links, oceanographic buoys, and alpine weather stations. A second strand involves fusing the experimental dataset with machine-learning models that recommend context-aware protocol stacks on the basis of environmental conditions, link budget, battery capacity, and the specific data-volume patterns typical of remote-sensing payloads. Third, coupling energy traces with life-cycle assessments that attach CO_2_-emission factors to each kilobyte of remotely sensed data would enable a genuinely sustainability-oriented comparison of competing RS-IoT architectures. Field validation inside operational remote-sensing infrastructures, e.g., wildfire-monitoring drone fleets or coastal radar buoys under real interference and variable ambient conditions, will be essential to confirm the external validity of laboratory findings. Finally, releasing an open repository that hosts raw power logs, interpolated RS-IoT traffic traces, and the accompanying scripts will enhance transparency and facilitate independent replication by the remote-sensing community.

Taken together, these research directions will transform the Unified Method into a domain-specific instrument for comparing communication protocols across heterogeneous remote-sensing deployments as well as under controlled laboratory conditions. The resulting roadmap will deepen analytical rigour and convert empirical insights into actionable engineering guidance, thus providing a robust foundation for selecting energy- and carbon-efficient communication technologies in both IoT/IIoT and remote-sensing projects.

## 6. Conclusions

We have presented an experimental and simulation evaluation of leading wired and wireless IoT/IIoT communication protocols regarding energy consumption, reliability, and maintainability. We combined heterogeneous laboratory measurements and simulations using a bilinear interpolation and weighted normalization scheme. We obtained a dimensionless energy efficiency index of 0–1. This index reveals a 50% difference between the most and least economical wired protocols, MQTT-SN at 58 J per 1 KB message and HTTP/3 at 108 J. At the same time, in the radio domain, BLE proves to be significantly more efficient than the energy-hungry Wi-SUN. Detailed analysis confirms that the combined cost of data transmission and idle current leakage outweighs all other factors affecting battery life. Optimizing these two components yields greater benefits than tuning radio parameters in isolation. Translating the benchmark into remote sensing, we introduced a scenario-based classification that maps each protocol to the practical realities of unmanned aerial vehicle (UAV) payloads, ocean buoys, remote weather stations, and dense urban sensor networks. Five engineering principles emerge from this mapping. Firstly, constrained edge devices must use CoAP Non-Confirmable or MQTT-SN, while other protocols, such as MQTT/TLS or HTTP/REST, should terminate at a network-powered gateway. Secondly, acknowledgements and high-quality of service (QoS) modes should be enabled only for emergency traffic. Omitting them from routine telemetry provides energy savings of 10–15%. Third, the choice of physical layer must be consistent with the range: sub-GHz LPWAN or cellular IoT becomes mandatory when distances exceed several kilometers. At the same time, short-range protocols such as BLE or ZigBee should reside in local mesh clusters or high-speed access points without overhead. Fourth, images or firmware of large data objects should be buffered and transmitted only when the broadband link and external power supply converge, while fixed, low-bandwidth links should only carry compact sensor frames. Finally, wherever a public LPWAN or mobile infrastructure is available, the transmission cost per byte, with aggressive tuning of eDRX/PSM timers, can be lower than with self-maintained repeaters, so leveraging existing networks is usually advantageous.

The Industrial Classification of Protocol Energy Profiles (ICoPEP) matrix, now expanded with remote-sensing scenarios, accelerates technology selection for Earth-observation deployments by embedding energy fingerprints in real-world constraints. In addition, we distil practical optimization tactics anomaly-driven sampling, selective QoS, the separation of lightweight telemetry from high-volume updates, and a “registration–range–protocol” triad for battery sizing showing, for example, that eliminating superfluous acknowledgements can extend node autonomy by up to 35%, while lengthening the sampling interval from one to ten seconds reduces average energy draw by roughly 60% under low-dynamics conditions.

The unified benchmark, the RS-aware ICoPEP matrix, and the accompanying design guidelines form a coherent and replicable framework for building energy-efficient, resilient, and long-lived RS-IoT architectures. By lowering both the total cost of ownership and the environmental footprint, these results lay a practical foundation for the next generation of sustainable remote-sensing systems.

## Figures and Tables

**Table 1:** Overview of representative IoT/IIoT protocols selected for energy evaluation.

Reference	Protocol	Full Name	Year of Standard	Type	General Characteristics
[5]	MQTT	Message Queuing Telemetry Transport	2013/2019	Wired/TCP	Lightweight pub/sub protocol, low overhead, widely adopted in industry
[6]	MQTT-SN	MQTT for Sensor Networks	2019 (OASIS contrib.)	Wireless/UDP	Optimized for constrained nodes, no TCP/IP required, topic registration
[7]	CoAP	Constrained Application Protocol	2014 (RFC 7252)	Wireless/UDP	REST-like, confirmable/non-confirmable modes, minimal headers
[5]	AMQP	Advanced Message Queuing Protocol	2012 (ISO/IEC 19464)	Wired/TCP	Transactional messaging, reliable delivery, complex framing
[8]	HTTP/1.1	Hypertext Transfer Protocol v1.1	1997 (RFC 2616)	Wired/TCP	Stateless client-server, large headers, widely supported
[9]	HTTP/2	Hypertext Transfer Protocol v2	2015 (RFC 7540)	Wired/TCP	Binary multiplexing, header compression, faster than HTTP/1.1
[10]	HTTP/3	Hypertext Transfer Protocol v3	2022 (RFC 9114)	Wired/UDP (QUIC)	QUIC transport, reduced latency, high encryption overhead
[11]	LoRaWAN	Long Range Wide Area Network	2015	Wireless/ LPWAN	Sub-GHz long-range, extremely low power, class-based operation
[8]	Sigfox	Sigfox Protocol	~2010	Wireless/LPWAN	Ultra-narrowband, small payloads, cloud-managed network
[7]	NB-IoT	Narrowband Internet of Things	2016 (3GPP Rel. 13)	Wireless/LTE	High coverage, deep sleep modes, 10+ years battery life possible
[12]	LTE-M	LTE for Machines (Cat-M1)	2016 (3GPP Rel. 13)	Wireless/LTE	Higher throughput than NB-IoT, mobility and voice support
[6]	BLE	Bluetooth Low Energy	2010 (BT 4.0)	Wireless/PAN	Short-range, mesh support; low latency, <10 mA TX peaks
[10]	ZigBee	ZigBee (IEEE 802.15.4)	2004	Wireless/PAN	Scalable mesh, very low standby current, mature industrial tools
[8]	Wi-Fi	Wireless Fidelity (IEEE 802.11)	1997+	Wireless/WLAN	High data rates, duty-cycling needed for battery operation
[9]	6LoWPAN	IPv6 over Low-Power Wireless PAN	2007 (RFC 4944)	Wireless/WPAN	IP-native addressing; integrates with CoAP, mesh capable
[11]	Wi-SUN	Wireless Smart Utility Network	2012+ (Wi-SUN FAN)	Wireless/Sub-GHz Mesh	High node count, self-healing mesh, used in utilities

**Table 2:** Key characteristics of selected wired communication protocols in terms of maintainability and reliability.

Protocol	Brief Maintenance & Reliability Description	Citation
MQTT	Standardized by OASIS; low implementation complexity and widely available libraries facilitate simple maintenance. Requires a broker, though the ecosystem is mature and well-documented. Minimal network overhead results in the lowest measured energy consumption with a cloud broker. Long-term studies confirm that QoS 1 saves ≈ 8% energy compared to HTTP.	[15,28]
MQTT-SN	Lightweight variant of MQTT optimized for non-TCP/IP sensor networks; eliminates the need for TCP/IP stack but requires a gateway to interoperate with classical MQTT, which introduces administrative complexity. Offers lightweight topic registration and gateway aggregation, extending MQTT benefits to ultra-constrained nodes.	[29]
CoAP	IETF RFC 7252 REST-compliant protocol; confirmable and non-confirmable modes allow designers to balance reliability against battery lifetime. Seamless integration with IP-based systems facilitates maintenance, while single-datagram payloads reduce fragmentation risks. Requires QoS monitoring to ensure consistent operation.	[30,31,32]
AMQP	ISO/IEC standardized protocol designed for transactional messaging with exactly-once delivery. Ensures high reliability and robust flow control, but large frame size and complex header structure increase administrative cost. Requires skilled maintenance of central brokers, making it best suited for gateways and mission-critical infrastructures.	[33,34]
HTTP 1.1/2/3	Ubiquitous client-server family; HTTP/2 and HTTP/3 introduce advanced requirements (TLS 1.3, QUIC monitoring) and remain easy to integrate with enterprise IT systems. However, large headers cause high energy consumption, especially on edge nodes. HTTP/3 reduces handshake latency but remains the most power-hungry option, complicating long-term maintenance in energy-constrained deployments.	[35,36]

**Table 3:** Key characteristics of selected wireless communication protocols in terms of maintainability and reliability.

Protocol	Brief Maintenance & Reliability Description	Citation
LoRaWAN	Brief maintenance & reliability description Chirp Spread Spectrum with adjustable spreading factors balances range against airtime; Class A gives the lowest energy draw. Long device lifetime (>10 years) with low maintenance of end-nodes. Requires upkeep of gateways and synchronization with network servers. AES-128 key management ensures security but adds administrative overhead.	[5]
DASH7	BLAST architecture with built-in multicast firmware update lowers service costs for large fleets. ≈30 µA average current makes it cost-effective for long-term deployments. Requires central server management and skilled integration.	[5]
Sigfox	Ultra-narrowband uplink (12-byte payload) with triple redundancy and AES-128. Operator-managed infrastructure minimizes maintenance needs on the user side. Very low throughput constrains applications but ensures predictable energy use and coverage.	[5]
NB-IoT	200 kHz LTE profile; eDRX ≤ 186 min and PSM ≤ 413 days extend field life beyond 10 years. Maintenance offloaded to operator (SIM provisioning, network management). OTA updates supported. Reliability depends on operator coverage.	[37]
LTE-M	LTE Cat-M1 (1.4 MHz) supports mobility and ≈300 kb/s DL. Compromise between energy budget and latency. Maintenance largely handled by operators, reducing user burden but increasing dependency.	[37]
BLE	40 channels at 2.4 GHz, mesh-capable. Low TX peaks (<10 mA) suitable for low-power sensing. Requires supervision of routing and firmware updates in larger networks. No central operator—maintenance responsibility lies with the owner.	[20]
ZigBee	IEEE 802.15.4 mesh/cluster-tree topology supporting up to 65k nodes. Mature management tools and self-healing reduce maintenance. Requires periodic firmware and encryption key updates.	[38]
Wi-Fi	High data rate, with modern “sleep-friendly” chipsets enabling duty-cycled operation. Requires careful power planning and ongoing maintenance of access points. High energy demand shortens device lifetime, raising service frequency.	[39]
6LoWPAN	IPv6 over 802.15.4 with routable addresses; no dedicated gateway needed. Facilitates unified network management but requires upkeep of border routers. Sensitive to routing inefficiencies in large deployments.	[12]
Wi-SUN FAN	Sub-GHz self-healing mesh with >95 M deployed nodes. Very high resilience but highest energy consumption among tested protocols. Requires advanced diagnostic tools to maintain. Widely used in smart metering and utility networks.	[12]
5G	Network slicing and URLLC profiles (<5 ms) provide deterministic latency. Extended DRX lowers idle drain, but wide bandwidth makes it the most energy-demanding cellular IoT option. Fully operator-managed, maintenance requirements.	[11]
LPWAN	Sub-GHz, ultra-narrowband links (e.g., LoRa, Sigfox) with star or star-of-stars topology. Payload ≤ 50 B, duty cycle < 1%. Long battery lifetime (>10 years) with minimal maintenance; private deployments require gateway upkeep, public ones depend on operator.	[37]

**Table 4:** Mapping of comparative methods.

EC	Method A	Method B	Method C	Method D	Method E
1	Physical, continuous *V* & *I* log (ms)	Physical, batched energy traces + PCAP	Simulation of radio duty-cycles	Physical, SBC power profiles	Multi-hop simulation
2	MQTT (QoS 0/1) vs. HTTP	MQTT, CoAP, AMQP, HTTP (1/2)	MQTT, MQTT-SN, CoAP, HTTP	HTTP 1/2/3	CoAP vs. MQTT-SN
3	100 sample × 84h, QoS, sampling rate	1000 packets, QoS, 16–1024 B	100 s, single payloads	256 kiB 8 MiB, assorted hosts	30 min, POST/PUBLISH traffic
4	*E* [J] + battery discharge curve	*E* [J] & J/s + packet volume	Mean power [mW]	*E* [J] per MiB	Cumulative energy over time
5	Long horizon Direct correlation with battery lifetime	Broadest protocol set Full network traffic captured	Only method covering MQTT-SN Full control of radio channel	Unique empirical data for HTTP/3	Accounts for RPL routing and network load
6	Restricted to MQTT vs. HTTP Single MCU platform	Lacks MQTT-SN, HTTP/3	Simulation no hardware artefacts	Confined to HTTP family	Covers only MQTT-SN & CoAP

**Table 5:** Mapping of evaluation methods (A–E) to protocols, data types, and key measurement aspects.

Method	Protocols	Data Types	Metrics Covered	QoS/Packet Size/Time Considered
A	MQTT, HTTP	Instantaneous and long-term measurements	energy, time	mainly MQTT
B	MQTT, CoAP, AMQP, HTTP	hardware data with various QoS/payload combinations	energy, packets	extensive
C	MQTT, MQTT-SN, CoAP, HTTP	simulation, average consumption	only energy	no packets, QoS
D	HTTP 1.1, HTTP 2, HTTP 3	client-to-cloud, SBC measurement	only energy	only HTTP
E	CoAP, MQTT-SN	simulation with routing	energy, number of messages	limited context

**Table 6:** Scoring of methods by evaluation criteria.

Method	Protocols	Data (Type)	Parameters	Total
A	1 pt	2 pt (physical)	1 pt	4
B	2 pt	2 pt (physical)	1 pt	5
C	2 pt	1 pt (simulation)	0 pt	3
D	1 pt	2 pt (physical)	0 pt	3
E	2 pt	1 pt (simulation)	0 pt	3

**Table 7:** Contribution of each method to the unified assessment model.

Method	Points	Percentage Contribution
A	4	(4/18) × 100 ≈ 22.2%
B	5	(5/18) × 100 ≈ 27.8%
C	3	(3/18) × 100 ≈ 16.7%
D	3	(3/18) × 100 ≈ 16.7%
E	3	(3/18) × 100 ≈ 16.7%

**Table 8:** Energy consumption for MQTT and HTTP.

Protocol	Version	*Pavg* [mW]	SD	E [J]	Battery Life [h]
MQTT	QoS 0	629.68	19.39	94.45	114.34
MQTT	QoS 1	614.33	22.25	92.15	117.20
HTTP	-	670.16	16.19	100.52	107.44

**Table 9:** Discharge of battery with MQTT QoS 0.

Time (h)	Discharge Theor. (%)	Discharge Real. (%)	Relative Error (%)
12	89	88	0.00
24	78	77	0.00
36	66	65	1.45
48	55	54	1.72
60	44	40	2.08
72	33	38	8.11
84	22	16	18.52

**Table 10:** Discharge of battery with MQTT QoS 1.

Time (h)	Discharge Theor. (%)	Discharge Real. (%)	Relative Error (%)
12	90	90	0.00
24	80	80	0.00
36	69	69	0.00
48	59	59	0.00
60	49	48	2.04
72	39	36	7.69
84	28	23	17.86

**Table 11:** Discharge of battery with HTTP.

Time (h)	Discharge Theor. (%)	Discharge Real. (%)	Relative Error (%)
12	89	88	1.12
24	78	77	1.28
36	66	65	1.52
48	55	54	1.82
60	44	40	9.09
72	33	38	15.15
84	22	16	27.27

**Table 12:** Theoretical energy consumption (Wh).

Time (h)	MQTT QoS 0	MQTT QoS 1	HTTP
12	7.56	7.37	8.04
24	15.11	14.74	16.08
36	22.67	22.12	24.13
48	30.22	29.49	32.17
60	37.78	36.86	40.21
72	45.34	44.23	48.25
84	52.89	51.60	56.29

**Table 13:** Energy consumption for sending 1000 B payload.

Protocol	Mode	Energy [J]	Time [s]	J/s
MQTT PUBLISH	QoS 0	74	45	1.6444
MQTT PUBLISH	QoS 1	76	48	1.5833
MQTT PUBLISH	QoS 2	80	50	1.6000
CoAP PUT	No confirm.	69	49	1.4082
CoAP PUT	Confirm.	69	45	1.5333
AMQP SEND	-	93	50	1.8600
HTTP POST	-	89	55	1.6100

**Table 14:** Energy consumption using COOJA simulator.

Protocol	P [mW]	E [J]
MQTT	1.00	0.15
MQTT-SN	0.80	0.12
CoAP	0.95	0.1425
HTTP	1.38	0.207

**Table 15:** Energy consumption (J) for SBC- Cloudflare and SBC-GCS.

Protocol	Version	SBC-Cloudflare Energy [J]	SBC-GCS Energy [J]
HTTP	1.1	12.13	9.914
HTTP	2	12.09	10.274
HTTP	3	15.75	11.526
HTTP	1.1	12.13	9.914

**Table 16:** Energy consumption (J) as a function of time simulation.

E [J]/t [min]	5	10	15	20	25	30
MQTT-SN	2.55	7.55	15.00	25.00	37.00	53.00
CoAP	2.50	7.50	15.05	25.10	40.00	57.00
MQTT-SN	2.55	7.55	15.00	25.00	37.00	53.00
CoAP	2.50	7.50	15.05	25.10	40.00	57.00

**Table 17:** Energy consumption (Econs.) of all studied protocols regarding different methods.

Method		A	B	C	D	E
Protocol	Mode	Econs. [J]	Econs. [J]	Econs. [J]	Econs. [J]	Econs. [J]
MQTT	QoS 0	94.45	74	-		-
MQTT	QoS 1	92.15	76	-	-	-
MQTT	QoS 2	-	80	-	-	-
MQTT-SN	-	-	-	0.12	-	53
CoAP	No confirm	-	69	0.1425	-	57
CoAP	Confirm	-	69	-	-	-
AMQP	-	-	93	-	-	-
HTTP1	-	100.52	89	0.207	12.13	-
HTTP2	-	-	83	-	12.09	-
HTTP3	-	-		-	15.75	-

values harmonized and missing entries estimated using bilinear interpolation.

**Table 18:** Ranking of wired protocols with unified method.

Protocol	Mode	Econs. [J]	Rank
MQTT-SN	-	58	1
CoAP	Non-confirmable	69	2/3
CoAP	Confirmation	69	2/3
MQTT	QoS 0	74	4
MQTT	QoS 1	76	5
MQTT	QoS 2	80	6
HTTP2	-	83	7
HTTP1	-	89	8
AMQP	-	93	9
HTTP3	-	108	10

values normalized and unified via bilinear interpolation.

**Table 19:** Protocol suitability for typical RS-IoT deployment scenarios.

	UAV-Based Payloads	Floating Buoys	Remote Met Stations	Urban Fixed Sensors
MQTT	A (with cellular/LTE-M)	U (no IP infra)	U (no IP infra)	A (if power/coverage)
MQTT-SN	P (if gateway present)	A (needs gateway)	P (via LPWAN gateway)	A (with gateway)
CoAP	P (efficient uplink)	P (with LPWAN)	P (with LPWAN)	P (for IP-based nodes)
AMQP	U (too heavy)	U (too heavy)	U (too heavy)	U (rarely justified)
HTTP/1.1	U (high overhead)	U (high overhead)	U (high overhead)	A (if mains power)
HTTP/2	U (overkill for UAV)	U	U	A (if mains power)
HTTP/3	U (overkill, high energy)	U	U	U (rarely used on sensor)
BLE (Bluetooth)	U (range too short)	U (range too short)	U (range too short)	P (short-range, low power, e.g., indoor)
ZigBee	U (no relay infrastructure in air)	U (range too short)	A (if multi-node mesh to gateway)	P (mesh clusters in city, if powered coordinator)
6LoWPAN	U (no infra aloft)	U (not typical)	A (for local mesh cluster)	A (used in smart city mesh with gateway)
Wi-SUN	U (mesh not feasible aloft)	U (no mesh infra)	U (unlikely, high power)	A (utility mesh with mains-powered routers)
LoRaWAN	P (long-range telemetry)	P (primary choice)	P (primary choice)	A (urban LPWAN networks)
Sigfox	A (if small data, limited ack)	P (if coverage exists)	P (if coverage, very low data)	A (urban if network available)
NB-IoT	P (if network available)	A (near coast or with coverage)	P (if network available)	P (leverages cellular coverage)
LTE-M	P (for high-data needs)	U (rarely in open ocean)	A (if moderate data, coverage)	P (for mobile/urban sensors)
Wi-Fi	A (for offloading large data when near base)	U (not feasible offshore)	U (no infrastructure)	P (if sensor has power & Wi-Fi AP)

## Data Availability

The data presented in this study are available on reasonable request from the corresponding author.

## Abbreviations

The following abbreviations are used in this manuscript:

UAV	Unmanned Aerial Vehicle
IoT	Internet of Things
IIoT	Industrial Internet of Things
SBC	Single Board Computer
QoS	Quality of Service
MQTT	Message Queuing Telemetry Transport
MQTT-SN	MQTT for Sensor Networks
CoAP	Constrained Application Protocol
AMQP	Advanced Message Queuing Protocol
HTTP/1.1, HTTP/2, HTTP/3	Versions of Hypertext Transfer Protocol
BLE	Bluetooth Low Energy
Wi-SUN	Wireless Smart Utility Network
6LoWPAN	IPv6 over Low power Wireless Personal Area Networks
NB-IoT	Narrowband Internet of Things
LTE-M	Long Term Evolution for Machines
LoRaWAN	Long Range Wide Area Network
PSM	Power Saving Mode
eDRX	Extended Discontinuous Reception
URLLC	Ultra-Reliable Low-Latency Communication
LPWAN	Low Power Wide Area Network
RIS	Reconfigurable Intelligent Surface
RRC	Radio Resource Control
RPL	Routing Protocol for Low-Power and Lossy Networks
OSCORE	Object Security for Constrained RESTful Environments
DTLS	Datagram Transport Layer Security
TLS	Transport Layer Security
AES	Advanced Encryption Standard
BCap	Battery capacity (Wh or %)
P(t)	Instantaneous power at time *t*
Pavg	Average power consumption (mW)
Pcons.	Average consumption power
E	Total energy consumed (Joules or Wh)
SD	Standard deviation
texec	Execution time of operation
Rt	Resistance (if applicable, but in your context: possibly symbolic)
ρ	Spearman rank correlation coefficient
BLife	Estimated battery lifetime (hours)
Econs.	Energy consumption (J)
ICoPEP	Industrial Classification of Protocol Energy Profiles
